# A Carbon Nanocomposite Material Used in the Physical Modelling of the Overburden Subsidence Process

**DOI:** 10.3390/nano13222962

**Published:** 2023-11-16

**Authors:** Jianlin Xie, Shan Ning, Qingdong Qu, Weibing Zhu, Bozhi Zhao, Jialin Xu

**Affiliations:** 1 State Key Laboratory for Fine Exploration and Intelligent Development of Coal Resources, China University of Mining and Technology, Xuzhou 221116, China; 2 School of Mines, China University of Mining and Technology, Xuzhou 221116, China; ts22020070a31ld@cumt.edu.cn; 3 CSIRO Mineral Resources, 1 Technology Court, Pullenvale, Brisbane 4069, Australia

**Keywords:** massive sandstone, ultra-thick key stratum, strata movement, mining engineering, carbon nanocomposite material

## Abstract

Carbon nanomaterial is widely used in structural health monitoring due to the advantage of sensitivity and good mechanical properties. This study presents a novel approach employing carbon nanocomposite materials (CNMs) to characterize deformation and damage evolution in physical modelling. As the primary measurement method, the CNM is used to investigate the deformation characteristics of a 200–400 m thick sandstone bed at a 1 km deep longwall mine. The sandstone unit is identified as an ultra-thick key stratum (UTKS), with its thicknesses varying across different mining panels of the UTKS. The results of CNM monitoring show that the UTKS remains stable even after a consecutive excavation of 900 m in width. This stability impedes the upward propagation of overlying strata failure, leading to minimal surface subsidence. The study demonstrates the huge potential of CNM in the mining area, which can be useful for investigating material damage in physical modelling studies. The findings suggest that the cumulative extraction width in individual mining areas of the mine should be controlled to avoid a sudden collapse of the UTKS, and that special attention should be paid to where the UTKS’s thickness changes substantially. The substantial variation in UTKS thickness significantly impacts the pattern of overburden subsidence.

## 1. Introduction

Geological conditions significantly impact ground behaviours in mining and civil engineering [1] and can lead to safety risks [2]. Typically, the overburden strata contain one or multiple thick, strong geological layers [3]. Recognising their significant role in governing the overburden subsidence and movement processes, researchers commonly define such competent geological layers as a key stratum (KS) [4,5]. KSs can vary in thickness, with a typical range of 10–50 m. Some coal mines may encounter an exceptionally thick KS with a thickness exceeding 100 m. Such a thick KS can be referred to as an ultra-thick key stratum (UTKS) [4]. 

Given that UTKS formations are composed predominantly of strong sandstone layers, mines encountering a UTKS have increasingly been the sites of high-energy microseismic events and dynamic failures [6,7]. Therefore, it is critical to understand the deformational behaviours of the UTKS and its impact on the overburden subsidence process at coal mines where site geological conditions differ. Due to its substantial thickness, the UTKS behaves differently in deformation and failure processes from a typical KS with an average thickness. The traditional beam theory may not be suitable for the UTKS [8,9]. Previous theoretical analyses and field measurements [10,11] have reported a distinct surface subsidence scenario: the subsidence coefficient in mines with a UTKS is significantly small. As the UTKS is capable of sustaining overburden load over a large span, a sudden collapse can lead to severe dynamic failures [12]. Notwithstanding these findings, the deformation process and failure mechanisms of the UTKS are poorly investigated compared to the KS with typical thicknesses. 

To investigate the deformational behaviours of geological formations, researchers employ various methods, including theoretical analysis, numerical simulation, field measurement, and physical simulation [13]. Each method has its own advantages and limitations. Physical simulation, for instance, provides a comprehensive representation of overburden deformation and strata movement, enabling back-analysis and geologic parametric studies to optimize underground engineering design [14]. While physical simulations have been extensively used to study overburden strata deformation in longwall mining with varying geological features, accurately measuring damage and micro-fractures remains a challenge [15].

Carbon nanomaterial, extensively used in structural health monitoring to assess damage and micro-fractures, forms a conductive network that alters resistance upon fracturing [16,17]. Notably, resistance escalates when the network sustains damage from fractures, particularly fibre fractures under tensile stress [18,19,20]. Demonstrating its successful application, a cement-based sensor incorporating this nanomaterial is extensively used in the construction industry. Many engineering [21,22,23] examples have proven the feasibility of integrating carbon nanomaterial into cement for damage and micro-fracture measurement. Consequently, this approach can also be employed in physical simulation experiments for similar measurements.

This study employs physical simulation as the primary method to investigate the deformation characteristics of a massive sandstone bed with a thickness of 200–400 m at a deep longwall mine in China. A large-scale physical model consisting of eight longwall panels was constructed for this purpose. In addition to the conventional photogrammetry technique for monitoring material deformation, a novel approach of using carbon nanocomposite material (CNM) was employed to characterise material damage by measuring changes in resistance. It is worth noting that this is the first instance of using CNM in this type of physical simulation study in underground mining engineering.

## 2. Geological Condition of the Study Mine

The study mine (Gaojiapu Coal Mine) is in the Changwu County of Shaanxi Province, China. It has a designed production capacity of 5 Mt/a. The mine extracts coal from the No. 4 seam with an 800–1000 m depth of cover. The seam is relatively stable, with an average thickness of 10.5 m and a maximum thickness of 15.75 m. The coal seam is assessed to be prone to coal bursts.

**Figure 1:**
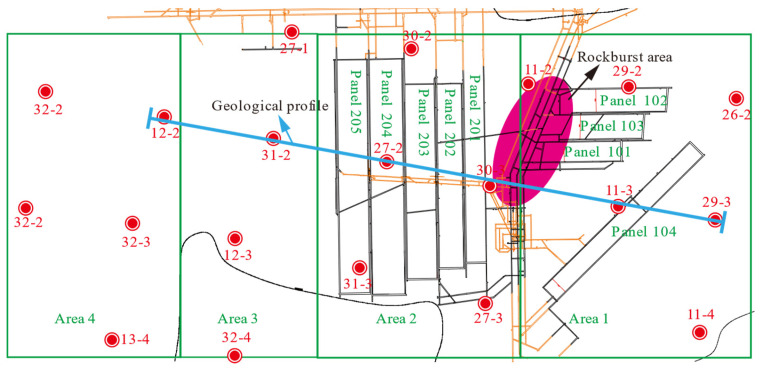
Layout of Gaojipu coal mine. The red-shaded areas underwent several dynamic events during the extraction of the 101–103 panels of Area 1.

The mine is divided into several areas for exploitation. Area 1, consisting of longwalls 101 to 103, was mined between 2015 and 2017. Area 2, comprising longwall 201 to 205, was mined between 2016 and 2021. During the excavation of Area 1, multiple dynamic events occurred on the main roads between Areas 1 and 2 (Figure 1).

Coal extraction from Longwall 101 to 205 resulted in minor surface subsidence. The maximum subsidence in Area 2 was measured at 443 millimetres, only about 0.044 times the extraction thickness (10 m on average). This subsidence coefficient is significantly smaller than that measured at an adjacent coal mine, which was about 0.65 [4].

**Figure 2:**
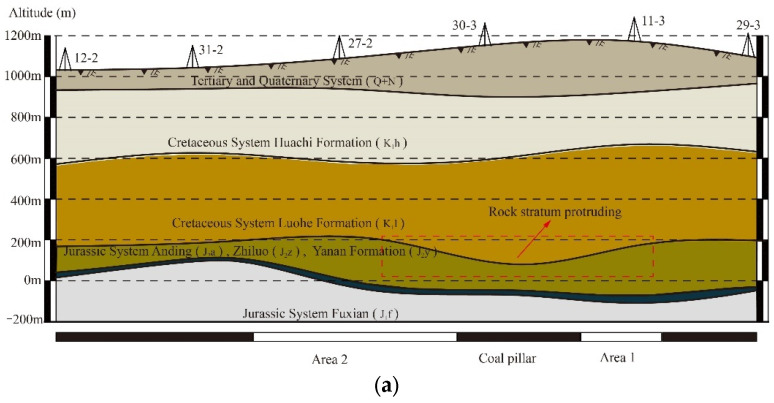

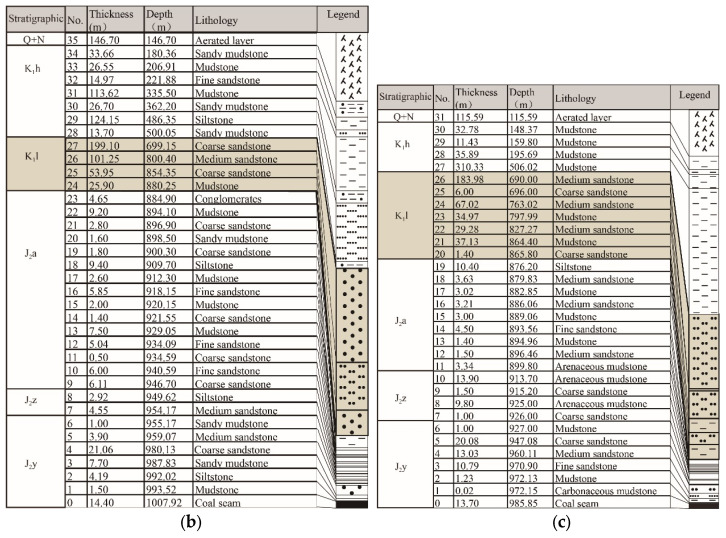
Gaojiapu Mine stratigraphic distribution profile. (**a**) Geological profile. (**b**) Borehole 27-2. (**c**) Borehole 30-3.

Figure 2a displays the stratigraphic profile of the study mine, generated from the logging data from the exploration holes 12-2, 31-2, 27-2, 30-3, 11-3, and 29-3. Figure 2b,c present the stratigraphic columns of two of the boreholes. The Luohe Formation (K_1_1) is composed of sandstones with a total thickness of over 100 m. The entire thickness of the Luohe Formation varies across different panels, forming a thickened zone over the pillar between Areas 1 and 2. The KSPBV5.0 software was used to identify the key stratum units, and the result suggests that the Luohe formation is a UTKS.

## 3. The Physical Model

### 3.1. Experimental Scheme

**Figure 3:**
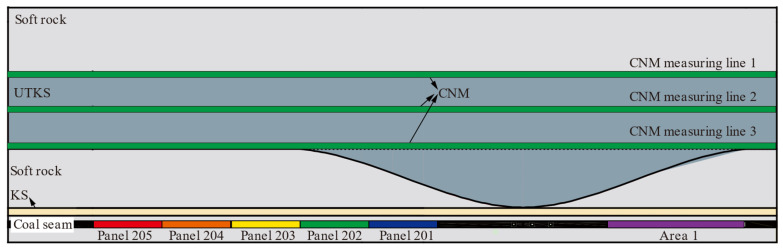
Model laying scheme.

A relatively large physical model 5.0 m wide and 1.66 m high was designed and constructed to simulate the deformation characteristics of the UTKS. The overlying strata were simplified, as shown in Figure 3. The thickness variation of the UTKS was considered in the model based on the thickness contour shown in Figure 2a. Model similarity ratio is a key factor in the design of the model parameters. Previously successful physical simulations have provided good references for selecting proper ratios. Based on the experience of [24], the geometric, stress, and density similarity ratios were determined as 1:400, 1:480, and 1:1.2, respectively.

The materials used to simulate rock layers consisted of calcium carbonate, gypsum, and river sand. The strength of these simulation materials was determined in correlation with the proportions of the material components. Mechanical test results from exploration drilling samples (as presented in Table 1) demonstrated that the Luohe formation (K_1_l) exhibits distinctively higher strength than other formations in UCS and tensile strength. The overburden was represented by four different types of rocks, namely, weak, UTKS, sub-key stratum, and coal seam. The details regarding the thickness and ratios of each layer can be found in Table 2.

The simulated coal seam was excavated following the sequence: Area 1 → Panel 201 → Panel 202 → Panel 203 → Panel 204 → Panel 205. The total excavation length was 315 cm, of which panels 201–205 were 45 cm each, and Area 1 was 90 cm. The excavation rate was 5 cm/hour to ensure the overburden deformation reached equilibrium.

### 3.2. The Conventional Monitoring System of Deformation

**Figure 4:**
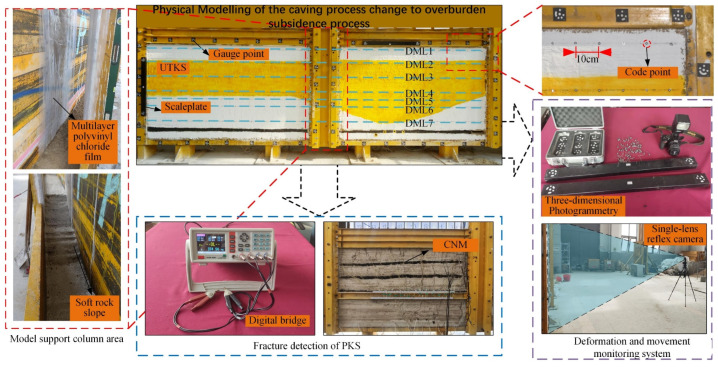
The experiment model and monitoring system. It is noted that a baffle plate was installed in the middle of the model to prevent a collapse of the 5 m wide model. Two thin layers of polyvinyl chloride film smeared with mechanical lubricants were placed between the plate and the model to reduce frictional forces.

The final model and the monitoring system are exhibited in Figure 4. The overburden deformation during the mining process was monitored with the CoordMeasis photogrammetry system, which supports 2D and 3D measurements and has a measurement accuracy of ≤0.10 mm. The system comprises a 24-megapixel SLR Nikon camera, code points, and a click ruler. Seven displacement measuring lines (numbered DML1–DML7) were arranged on different levels of the model. The displacement of each measuring point was calculated after each excavation.

Before excavation, code points were laid horizontally with a spacing of 10 m on the exterior surface of the model. Gauge points were arranged vertically on the measurement line to mark the monitoring points. Rulers and code points were placed on the frame around the model. These code points were fixed, serving as measurement benchmarks.

## 4. Using Carbon Nanocomposite Material (CNM) to Characterise Damage

To enhance the characterisation of the UTKS deformation characteristics, we introduced a novel approach of using highly conductive CNM to reflect material deformation characteristics by measuring material resistance changes. Applications of similar materials in concrete deformations [24,25] have demonstrated their sensitivity to deformation and damage.

### 4.1. Preparation of the CNM

**Figure 5:**
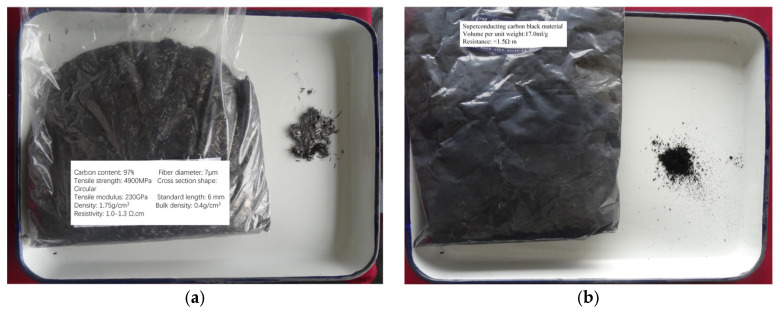
The CNM materials: (**a**) the chopped carbon fibre sourced from Toray Industries, Inc. (Tokyo, Japan); and (**b**) the nano-carbon black sourced from Suqian Xigu Nanotechnology Co., Ltd. (Suqian, China).

The CNM was composed of 6 mm long chopped carbon fibre (Figure 5a) and nano-carbon black (Figure 5b). The two materials have a resistance of approximately 0.15 Ω·m and 1.5 Ω·m, respectively. Drawing from a study [26] carried out on concrete specimens, which indicated that a minimal weight content of carbon fibre had little to no effect on the strength of the material and that a content exceeding 1.5% would demonstrate sufficient sensitivity to deformation, the weight content of carbon fibre materials in this study was determined as 2%.

The CNM was prepared in several steps. First, the chopped carbon fibre, nano-carbon black, and water were mixed using a high-speed mixer. Next, the mixture was transferred into another mixer, where they were evenly blended with gypsum, calcium carbonate, and river sand for 3 min. Subsequently, a designed amount of water was added, and the mixing continued for another 3 min.

### 4.2. Resistance Change of CNM during Model Settlement

**Figure 6:**
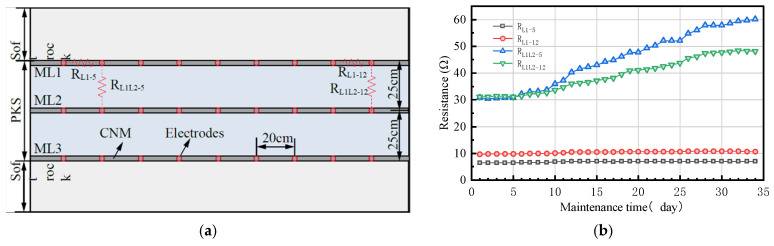
Variation in resistance during the settlement period of the materials. (**a**) Scheme of CNM laying and measurement. (**b**) Variation in resistance during the maintenance period of simulation materials.

Three layers of 2 cm thick CNM were laid in the top, middle, and bottom sections of the UTKS, respectively (Figure 3 and Figure 6a). The resistance of a CNM layer and the conventional material were measured during the settlement process of the model. The monitoring instrument used is a Victor LCR digital bridge (VC4090A from Yisheng Victor Tech Co., Ltd (Shenzhen, China), accuracy 0.05%). Figure 6b presents the results, which show that the resistance of the conventional material significantly increased as the model settled down, whereas the resistance of the CNM was almost unchanged. The results demonstrate the high conductivity of the CNM compared to the conventional materials and a negligible impact of moisture loss.

### 4.3. Using Resistance Change to Reflect Material Damage

**Figure 7:**
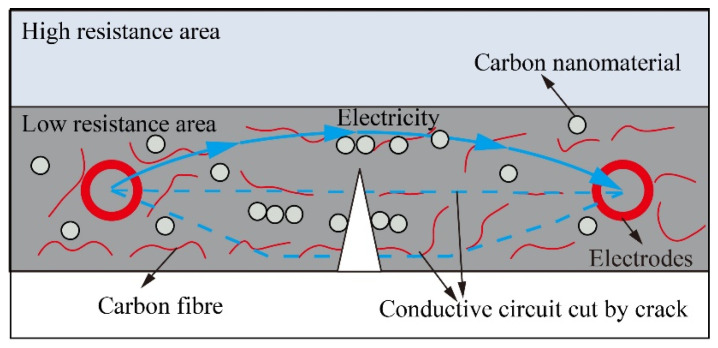
Schematic diagram of the conductive circuit with a crack formed in the bottom of the CNM.

Various studies [25,27] have suggested that the electrical resistance of the CNM changes as the material experiences damage. This correlation leads to the idea of incorporating CNM in the simulated rock layers to measure their electrical resistance changes as a result of excavation. In the physical model, the buried CNM layers would form the primary circuit of conduction [28]. When a crack forms in a layer (as illustrated in Figure 7), the connection between carbon nanomaterials will disengage, and the carbon fibre will fracture. This leads to the conductive circuit being partially or entirely cut off, increasing the resistance of the CNM [29,30]. In other words, increasing resistance reflects certain damage to the material.

As a longwall face advances, its overlying strata behind the longwall face generally undergo bending deformation, with the lower section fractured and caved. Under such deformation modes, the electrical resistance on different levels and at different times would experience various patterns such as gradual increase, abrupt increase, and stabilisation. These changing patterns, which can be measured, are indicative of distinct characteristics of deformation [31]. For instance, an abrupt increase in resistance would mean significant damage to the conductive network [26,32].

To characterise the evolution of the UTKS’s damage in this modelling study, a parameter named resistance change ratio (*RCR*) of the CNM is defined in this paper and is expressed below.

(1) $$RCR=\frac{R_{t\;}-R_{0}}{R_{0}}\times 100\%$$

where *R_t_* represents the measured resistance during excavation, and *R*_0_ is the initial resistance before the excavation.

## 5. Physical Simulation Results

### 5.1. Overburden Subsidence Process

**Figure 8:**
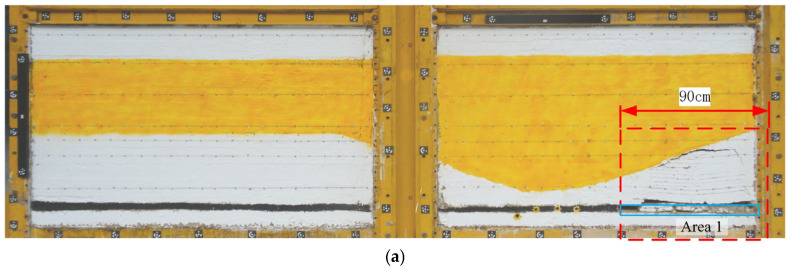

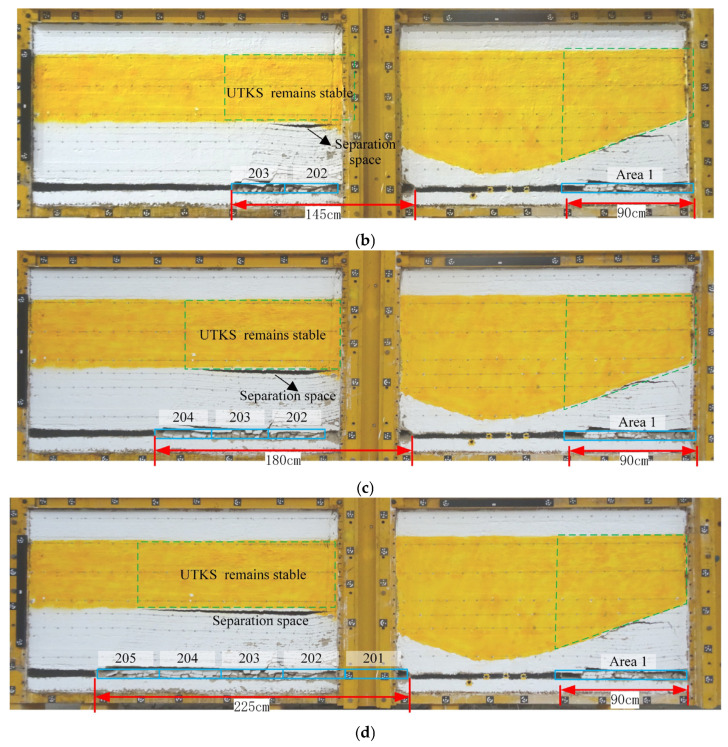
The simulated caving and fracturing process of overburden. (**a**) After the excavation of Area 1. (**b**) After the excavation of Panel 203. (**c**) When mining to the Panel 204 working face. (**d**) When the mining of Panel 205 ends.

Figure 8 shows the simulation results during the excavation process, starting from Area 1 to the five panels in Area 2 (201–205). These results reflect overburden subsidence development. The immediate roof, which is a weak rock unit, caves immediately following the extraction of each panel. Above the immediate roof, mining-induced fractures extend up to the bottom of the UTKS. Significant bedding separation is formed beneath the UTKS in the Area 2 panels, with its lateral extent increasing as excavation continues. No fractures can be visually observed within and above the UTKS.

The extended bedding separation in Area 2 led to the UTKS overhanging over a large span of up to 900 m (approximately 225 cm in the model at a 1:400 scale) without losing stability. The stable UTKS impeded further propagation of strata fracturing, resulting in the minor subsidence of its overlying strata.

### 5.2. Deformation of the Overlying Strata on Different Levels

**Figure 9:**
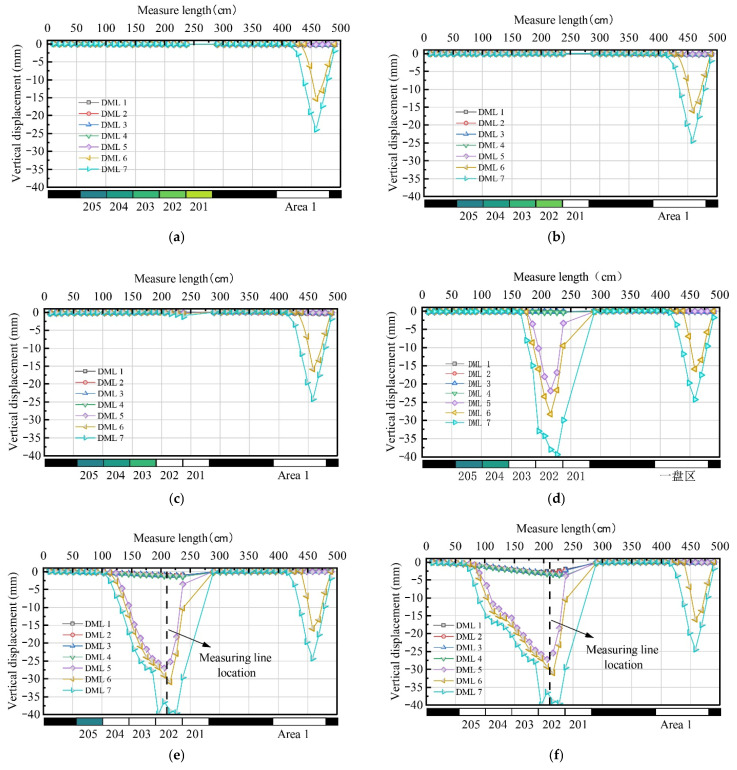
Subsidence of measuring lines after mining of each panel. (**a**) When the mining of Area 1 ends; (**b**) when the mining of Panel 201 ends; (**c**) when the mining of Panel 202 ends; (**d**) when the mining of Panel 203 ends; (**e**) when the mining of Panel 204 ends; and (**f**) when the mining of Panel 205 ends.

The vertical displacement measured by various measurement lines during the excavation is depicted in Figure 9. The results show that, after the extraction of Areas 1 and 2, the KS1 and the weak strata (reflected by line DML 5–7) sag significantly, whereas the UTKS (reflected by line DML 2–4) undergoes only minor displacement.

After all panels are excavated (Figure 9f), the maximum displacements on DMLs 5, 6, and 7 are −27.24 mm, −31.05 mm, and −39.99 mm, or 68.10%, 77.7%, and 99.8% of the extraction thickness (40 mm), respectively. In contrast, the maximum subsidence values of DMLs 1, 2, 3, and 4 within and above the UTKS are only −2.86 mm, −3.10 mm, −3.50 mm, and −3.65mm, or 7.2%, 7.8%, 8.8%, and 9.2% of the extraction thickness, respectively. The significant difference in subsidence leads to the large separation beneath the UTKS (up to 23.90 mm) and a small subsidence coefficient on the top of the model (only 0.072 mm).

**Figure 10:**
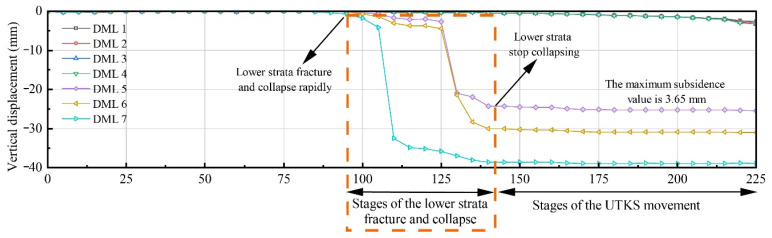
Strata movement in the middle of Panel 202.

Figure 9 also suggests that the overlying strata near Panel 202 undergo the most significant displacement. To further analyse overburden movement, the subsidence value at the middle point of Panel 202 (Figure 9e,f) on various measurement lines was extracted and plotted in Figure 10. 

### 5.3. Deformation Distribution within the UTKS

**Figure 11:**
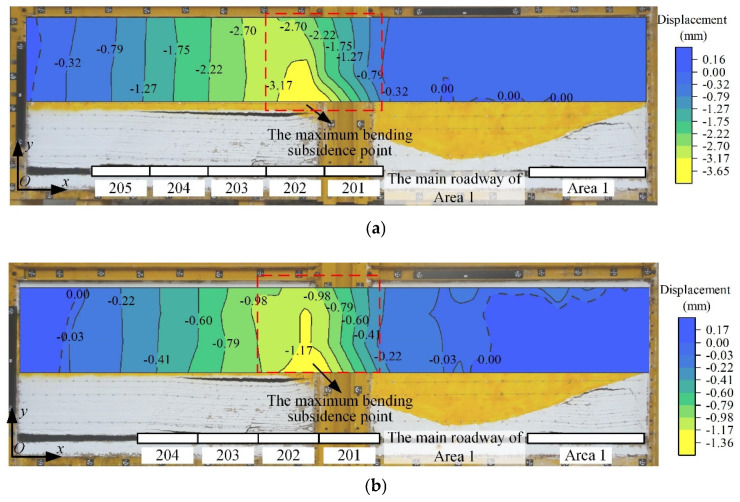

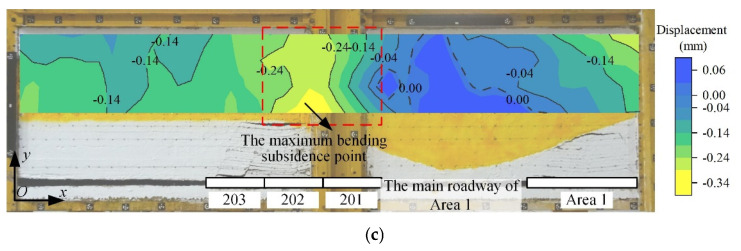
Analysed distribution of displacement in the UTKS during excavation. (**a**) Displacement distribution in the UTKS after Panel 203 was excavated. (**b**) Displacement distribution in the UTKS after Panel 204 was excavated. (**c**) Displacement distribution in the UTKS after Panel 205 was excavated.

Utilizing MATLAB R2021b surface interpolation, the displacement distribution within the UTKS was extrapolated to a contour format, as shown in Figure 11. The results show that minor deformation in the UTKS occurs, and the deformation values vary horizontally and vertically. The area above Panels 201 and 202 exhibits the maximum deformation, and a noticeable relationship emerges between the location of the maximum deformation and the change in UTKS thickness, which increases towards the area pillar from Panels 201 and 202. In the area spanning Panels 203 to 205, the deformation remains consistent vertically due to the uniform thickness of the UTKS, but it exhibits variation horizontally.

### 5.4. Damage Distribution within the UTKS

**Figure 12:**
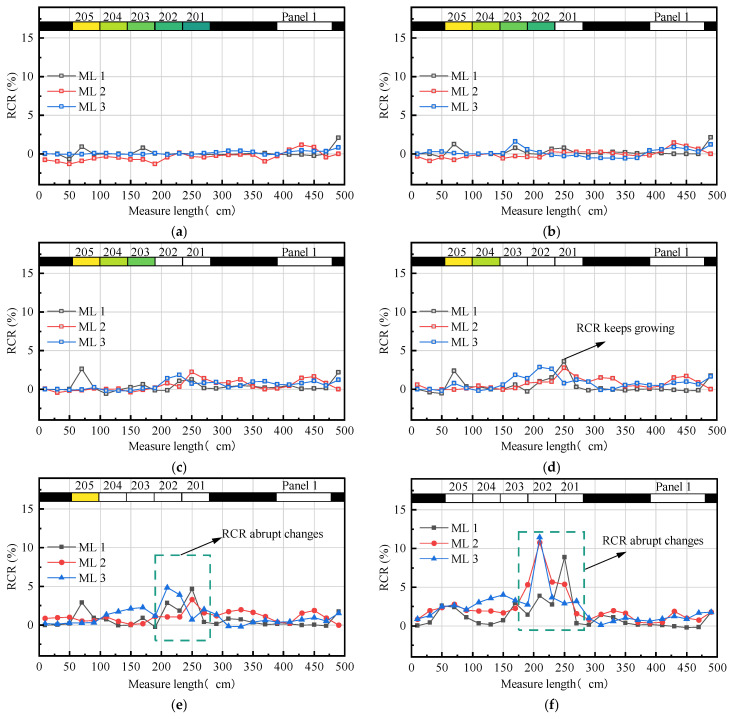
Variations of *RCR* under different mining conditions. (**a**) When the mining of Area 1 ends; (**b**) when the mining of Panel 201 ends; (**c**) when the mining of Panel 202 ends; (**d**) when the mining of Panel 203 ends; (**e**) when the mining of Panel 204 ends; and (**f**) when the mining of Panel 205 ends.

As discussed in Section 4.3, changes in material resistance can reflect the level of material damage. After each excavation, the resistance of each part was measured, and the damage index *RCR* was calculated. Figure 12 shows the results.

In Section 4.3, we discussed how changes in material resistance can indicate material damage levels. We measured resistance after each excavation and calculated the damage index RCR (see Figure 12). The results reveal that RCR at each point gradually increases as the excavation progresses, and the RCR values differ at different measurement points. The highest RCR value appears in the area above Panels 201 and 202 (Figure 12f). Before the excavation of these two panels (Figure 12a,b), the *RCR* of these three layers had little changes. However, after these two panels were excavated (Figure 12d), the *RCR* in the area above 201 and 202 increased quickly to 3.60% and 2.83%. The two values further increased with the excavation of Panels 204 and 205 (Figure 12e,f), and eventually peaked at 4.86% and 11.44%, respectively. The relatively high *RCR* value above the Panels 201 and 202 area suggests that the UTKS experienced significantly more damage than in other zones.

**Figure 13:**
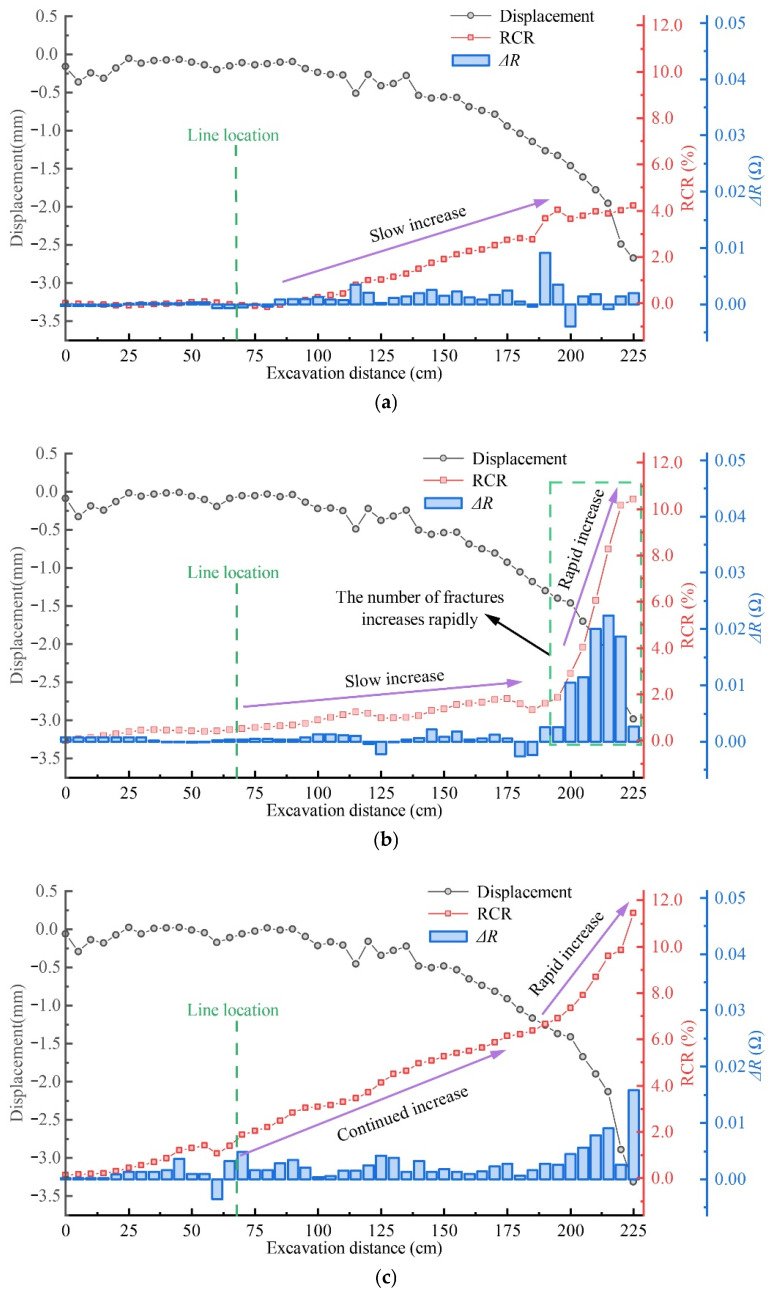
Displacement, *RCR*, and resistance difference change curves of UTKS during coal seam mining. (**a**) Variation of *RCR* of the top interface (ML 1) of the key stratum. (**b**) Variation of *RCR* of the middle interface (ML 2) of the key stratum. (**c**) Variation of *RCR* of the bottom interface (ML 3) of the key stratum.

To better understand the damage evolution, Figure 13 shows the evolution of the *RCR* value at the middle point of the three CNM layers. The figures also compare RCR to the displacement and the resistance change rate (Δ*R*), a parameter calculated as the difference between every two consecutive measurements.

Figure 13 clearly shows that the change in *RCR* values correlates with the change in displacement. After the excavation has passed the measuring point, both displacement and *RCR* show an increasing trend. The top line has the lowest maximum value (4.21%), whereas the middle and bottom lines are significantly larger, about 10.42% and 11.44%, respectively.

Δ*R* appears to be a good indicator of damage evolution. For ML 2, Δ*R* reached the maximum (0.022) when the excavation was 215 cm wide. The value is approximately eight times the average value (0.003) of ML 2 during the entire excavation process. While the displacement data shows no discernible differences between different measurement lines of the UTKS (Figure 9 and Figure 10), the *RCR* values on these three lines clearly differ.

## 6. Further Analysis with Analytical Models

### 6.1. Influence of UTKS Thickness and Span on Its Deformation

The primary objective of this study was to understand the impact of UTKS thickness and span on its deformation and subsidence. This analysis revealed that the subsidence caused by UTKS is relatively minor, corroborating the findings of numerous prior studies [33,34] that have emphasized the significance of geological layer thickness in affecting the subsidence process. To further analyse the influence of KS thickness and the excavation width on its deformation, the deflection of a fixed beam model was analysed using elasticity theory and the finite element method (FEM) [35]. The deflection is solved with a simplified formulation expressed in Equation (2).

(2) $$\vartheta =\frac{l^{4}}{2E}(\frac{\rho g}{h^{2}}+\frac{q}{h^{3}})$$

where ν is the beam deflection at the point *x* = 0, *y* = 0, *l* is the half beam span, *ρ* is the volumetric weight of the rock formation, *g* is the gravitational acceleration going, *q* is the overlying rock load, and *E* is the modulus of elasticity.

**Figure 14:**
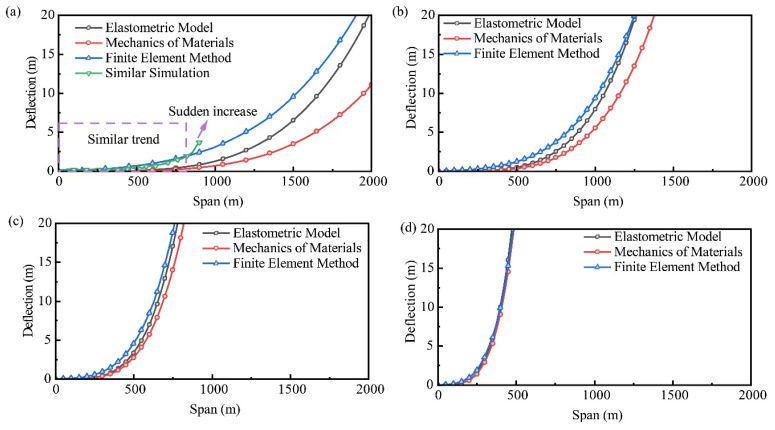
The deflection of different thickness UTKS: (**a**) 400 m; (**b**) 200 m; (**c**) 100 m; and (**d**) 50 m.

Figure 14 shows the analysed results. With a thickness of 400 m (Figure 14a), the deflection is minimal at a span of 800 m. This theoretical analysis result is consistent with the experimental results shown in Section 4.2. It also agrees with the field survey data that have suggested a maximum surface subsidence of only 0.44 m after Panels 201–204 were mined out (the total excavation width is 720 m) [36].

The deflection gradually increases with increasing span. After reaching a span of approximately 1000 m, the deflection increases rapidly, indicating material damage [32,33]. Given that substantial damage in the UTKS can result in intensive seismicity, the total excavation width should be controlled to prevent the occurrence of dynamic hazards. Measures such as optimizing the mine layout, for example, maintaining wide pillars between mining areas to disrupt the subsidence effect, could prove beneficial.

A smaller thickness results in greater deflections at the same span and smaller critical spans, as shown in Figure 14a–d. For instance, when the thickness is 50 m (Figure 14d), the critical span reduces to 200 m only. This result suggests that special attention should be paid to regions where the thickness of the UTKS significantly changes.

### 6.2. The Influence of Varying Thickness on Deformation

Prior research [37] has emphasized the significance of surface geology and subsurface structures on deformation patterns. The physical modelling results suggest that the UTKS would experience an asymmetrical deformation across the excavated panels, with the maximum subsidence in the Panel 202 area. This finding contrasts the typical symmetrical deformation predicted by the beam model [38,39]. A likely cause of this discrepancy is the varying thickness of the UTKS.

**Figure 15:**
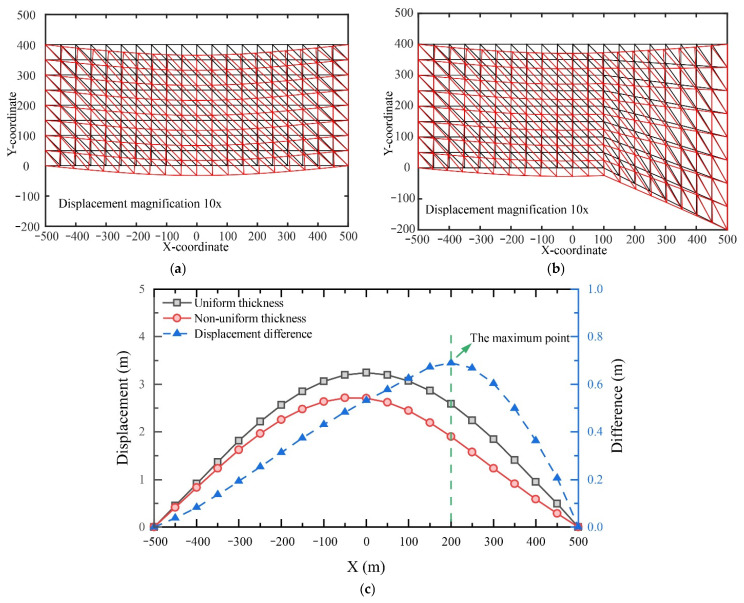
Analysed displacement of a thick beam: (**a**) with a uniform thickness; (**b**) with a varying thickness; (**c**) a comparison of the displacement on the bottom. (In (**a**,**b**), the grids in black lines show the initial model, and the grids in red lines are the analysed deformed model).

To illustrate this explanation, we established two FEM models, one having a uniform thickness (Figure 15a) and the other a varying thickness (Figure 15b). The latter has a thickness increasing from 100 to 500 m in the x-coordinate, representing the thickening part of the UTKS shown in the physical model.

Figure 15c compares the analysed deformation from these two models. The results show that the displacement of the uniform-thickness beam is symmetrical, whereas it is asymmetrical in the varying-thickness beam model. The increase in thickness results in an overall decrease in displacement. Comparing the two displacement curves, the greatest difference in displacement is located approximately 100 m from the thickness-changing point (X = 100 m). The deformation behaviour derived from these thick beam models is consistent with that obtained from the physical model, highlighting that special attention should be paid to regions where the thickness of the UTKS changes.

### 6.3. The Influence of Changing Thickness on the Location of UTKS Damage

Previous studies [40,41] suggest that a rock stratum analysed as a fixed beam generally starts to experience damage from the two fixed ends. However, the physical model shows that the UTKS experiences damage in the middle area of the excavation. Again, this difference may be attributed to the irregular shape of the UTKS. To further understand the damage characteristics of a UTKS with varying thickness, the von Mises theory [42], which uses the value of Von Mises stress (*σ*_mises_) to predict if a material will yield or fracture, is employed. Higher *σ*_mises_ values mean greater potential for material damage or failure [43,44].

**Figure 16:**
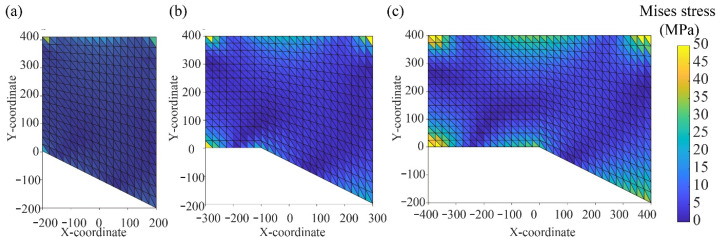
Distribution of *σ*_mises_ in three non-uniform thickness models: (**a**) with a span of 400 m; (**b**) with a span of 600 m; and (**c**) with a span of 800 m.

Figure 16 shows the contours of *σ*_mises_ for three models with different lengths. These models reflect the progressive excavation from Panel 201 to Panel 205 (Figure 3). All models show that the two fixed ends present larger *σ*_mises_ values than other parts of the beam, which agrees with the analysed damage pattern for a typical fixed beam model.

The *σ*_mises_ values in all three models exhibit an asymmetric distribution. At the early mining stage (Figure 16a), the left end, or the thinner end, presents the highest *σ*_mises_ values. Within the span extended under a uniform-thickness region (−300~−100 m, shown in Figure 16b), the *σ*_mises_ values at the thickness-changing point (−100 on the x-coordinate) become the largest except for the two ends of the fixed beam. Further increase in the span (further progression of the mining) results in increasing *σ*_mises_ values at the thickness-changing point. The results also show that the thicker section of the beam has lower *σ*_mises_ values than the thinner section. These observations explain the phenomenon observed in the physical model that the UTKS in the Panel 202 region, which is below the thickness-changing point of the UTKS, undergoes more significant damage than in other excavation areas.

The analysis clearly shows how variations in thickness impact the location of UTKS damage and deformation. These results support the results presented in Section 4.3, highlighting the significant sensitivity of carbon nanocomposite materials to deformation and damage. These findings might help us to design the prediction of surface subsidence and disaster risk with physical simulation. However, the analysis based on RCR needs more clarification, as the existing explanation is indistinct. Additional research is required to elucidate the mathematical relationship between deformation, damage, and RCR.

## 7. Conclusions

The presence of a massive, strong sandstone bed in a mining engineering context can pose significant threats to mining safety. To address this issue, CNM (carbon nanocomposite material) was introduced for the first time in this type of physical modelling method as an innovative approach to conventional deformation measurement methods, analysing material damage by measuring resistance changes. This is crucial for assessing and managing safety risks. Additionally, analytical models were established to enhance the understanding of the physical modelling results. The following conclusions can be drawn from this study.

1. The 200–400 m thick sandstone layer remains stable after a cumulative excavation width of 900 m. The stability prevents the upward propagation of strata failure, resulting in significant bedding separation underneath the sandstone layer and a minor surface subsidence of only 0.072% of the excavation thickness.
2. Although the thick sandstone layer remains unbroken, it undergoes continuous deformation as excavation progresses. Minor deformation is observed in the first 540 m excavation, which then evidently increases. The maximum deformation was found in the middle part of the total excavation.
3. The varying thickness of the thick sandstone layer shows a significant impact on the caving process and overall subsidence pattern. Both the physical and analytical models show that the thick rock layer experiences considerable damage in the thickness-changing region, emphasising the need for special attention to this area.
4. To mitigate risks associated with the thick sandstone layer, it is recommended that the mine should control the cumulative excavation width within a critical limit in each mining area. The continuous monitoring and assessment of the behaviour of the thick layer are also essential.
5. This study demonstrates that carbon nanocomposite materials’ high sensitivity to deformation and damage can be effectively tracked using RCR and Δ*R* values. The most pronounced damage, as indicated by the highest RCR values, occurs above Panels 201 and 202. Despite the uniformity in displacement data across different measurement lines, the combined use of RCR and Δ*R* offers a robust tool for evaluating damage evolution during excavation.

The physical model’s size limitation precluded reaching the eventual failure of the thick sandstone layer, making it impossible to assess the consequent failure. Future studies may benefit from employing a smaller geometry similarity ratio to simulate larger areas, especially in physical models with similar geological conditions. Carbon nanocomposite materials show potential for the physical modelling of overburden subsidence and damage. Further research is necessary for the specific methods employed for material formulations, elector layout, and other technology details. A deeper understanding of these details would enrich the study and serve as a guide for future research in the mining field.

## Figures and Tables

**Table 1:** Mechanical test results of geological drilling samples.

Stratigraphic Age	Lithology	Compressive Strength (MPa)	Tension Strength (MPa)	Young’s Modulus (GPa)	Poisson’s Ratio
		Min–Max	Min–Max	Min–Max	Min–Max
Huachi formation (K_1_h)	Mudstone	25.1–35.2	0.83–0.88	8.38	0.25
	Sandy mudstone	23.8–79.9	0.79–3.19	9.12–16.02	0.20–0.26
	Siltstone	58.3–72.8	2.85–3.04	13.16	0.18
	Fine sandstone	40.6–56.1	2.71–3.22	13.95	0.2
Luohe formation (K_1_l)	Sandy mudstone	58.2–68.9	2.14–2.20	11.04	0.19
	Siltstone	44.4–79.0	1.60–4.49	12.08–19.12	0.18–0.22
	Fine sandstone	31.2–62.6	2.13–3.60	9.57–18.23	0.17–0.23
	Medium sandstone	25.8–42.6	0.67–2.05	9.36	0.24
	Coarse sandstone	38.2–44.3	1.09–1.22	10.21	0.2
Anding formation (J_2_a)	Sandy mudstone	20.1–30.7	1.96–1.99	12.27	0.2
Zhiluo formation (J_2_z)	Siltstone	25.9–29.3	1.11–1.28	9.53	0.23

**Table 2:** Model laying scheme.

Rock Stratum	Thickness/m	Laying Thickness/cm	Ratio Number	Sand/kg	Calcium Carbonate/kg	Gypsum/kg
Weak rock	82.0	20.5 (2.0 cm each layer)	673	516.6	60.2	28.8
UTKS	200.0–440.0	50.0–88.0	437	1553.6	116.5	271.9
Weak rock	100.0	38.5 (2.0 cm each layer)	673	690.5	80.5	34.5
Sub-key stratum	20.0	5.0	455	126.0	7.5	17.6
Weak rock	14.0	3.5	673	88.2	10.2	4.4
Coal seam	16.0	4.0	773	100.8	10.1	4.3
Weak rock	40.0	10.0 (2.0 cm each layer)	673	252.0	29.4	12.6
Total	1047.0	166.0		3327.7	314.4	374.1

## Data Availability

The datasets used or analysed during the current study are available from the corresponding author upon reasonable request.
